# L_2,1_-GRMF: an improved graph regularized matrix factorization method to predict drug-target interactions

**DOI:** 10.1186/s12859-019-2768-7

**Published:** 2019-06-10

**Authors:** Zhen Cui, Ying-Lian Gao, Jin-Xing Liu, Ling-Yun Dai, Sha-Sha Yuan

**Affiliations:** 1 School of Information Science and Engineering, Qufu Normal University, Rizhao, China; 2 Library of Qufu Normal University, Qufu Normal University, Rizhao, China; 3 Co-Innovation Center for Information Supply & Assurance Technology, Anhui University, Hefei, China

**Keywords:** Drug-target interaction prediction, Graph regularization, L_2,1_-norm, Matrix factorization, Manifold learning

## Abstract

**Background:**

Predicting drug-target interactions is time-consuming and expensive. It is important to present the accuracy of the calculation method. There are many algorithms to predict global interactions, some of which use drug-target networks for prediction (ie, a bipartite graph of bound drug pairs and targets known to interact). Although these algorithms can predict some drug-target interactions to some extent, there is little effect for some new drugs or targets that have no known interaction.

**Results:**

Since the datasets are usually located at or near low-dimensional nonlinear manifolds, we propose an improved GRMF (graph regularized matrix factorization) method to learn these flow patterns in combination with the previous matrix-decomposition method. In addition, we use one of the pre-processing steps previously proposed to improve the accuracy of the prediction.

**Conclusions:**

Cross-validation is used to evaluate our method, and simulation experiments are used to predict new interactions. In most cases, our method is superior to other methods. Finally, some examples of new drugs and new targets are predicted by performing simulation experiments. And the improved GRMF method can better predict the remaining drug-target interactions.

## Background

With advances in drug discovery technologies, the existing methods can identify drug targets to some extent. But drug development is a high-cost, inefficient problem [1]. For drug developers, there has been a great deal of interest in the repositioning of drugs. This repositioning has some potential to reduce risk time and cost [2]. A crucial element for the repositioning of medicines is online biological databases such as KEGG [3], DrugBank [4], STITCH [5] and ChEMBL [6], which store a large number of current drug-target interactions. It is worth noting that there are still many interactions that have not been found [7]. Therefore, the advances of drug-target prediction technology is accelerated, and more and more prediction methods are proposed [8]. These computations, which reasonably predict new and unexplored interactions, have greatly facilitated the drug discovery process, making the process more credible. Recent research shows that there are three popular methods for predicting drug-target interactions, such as ligand-based methods [9], docking-based methods [10], and chemogenomic approaches [11]. Of course, we can also use the opposition-based learning particle swarm optimization to predict interactions, such as SNP-SNP interactions [12]. Moreover, the potential gene-gene interactions network can be identified by LNDriver [13].

Recently, many researchers have used matrix decomposition methods to solve drug-target interaction problems. The main methods are Bayesian matrix factorization, KBMF2K [14] and collaborative matrix factorization method, CMF [15]. A high-dimensional drug-target interaction matrix is decomposed into a plurality of low-dimensional matrices, and these matrices have characteristics of the original matrices, which is the principle of these methods. However, in theories, the above methods of matrix factorization still have some room for improvement. [16].

Using chemogenomic approaches to predict drug-target interactions is an effective method. The reason is that the first two methods have their own drawbacks. If a docking simulation is used, the three-dimensional structure of a target protein must be available. Furthermore, for ligand-based methods, if there are few or no target proteins known, this would be a problem that cannot be ignored. [9]. The advantage of using chemical genomics is that the information from the drugs and targets is used simultaneously for prediction [17]. New interactions are inferred by calculating the similarity of the chemical structures between drugs and the similarity of the genomic sequences between the targets. In this paper, the drug similarity and the target similarity are based on the construction methods in previous studies, which are based on the characteristics of the drug and the characteristics of the target. Its advantage is that we are better able to compare it with other methods, which is universal. However, if the same construction method of the drug similarity and the target similarity is used, this may affect the final results.

Two separate models are used to train drug target pairs, one based on the drug side and the other based on the target side. Thus, the final results are solved by predicting these two aspects. In this paper, to avoid over-fitting and sparing the target, the L_2,1_-norm is added in our method, which can eliminate some unattached target pairs [18]. Ten-fold cross-validation is used to evaluate the performance of our method.

We present the experimental results in Results. In Datasets, we conducted a case study. And we summarize this paper in Cross-validation experiments. In Interaction prediction under CVd, we clearly introduced the methods, including specific iteration formulas and algorithms.

## Results

### Datasets

Four datasets are used to experiment: the nuclear receptor (NR), the G protein-coupled receptor (GPCR), the ion channel (IC) and the enzyme (E). The size of these four datasets is different. Nuclear receptors are one of the most abundant transcriptional regulators in metazoans. NR includes some steroid hormones, vitamin D and quinone. In recent years, nuclear receptors have received widespread attention. For example, they are closely related to the development of diseases such as diabetes and fatty liver. Among them, PPAR-g agonist thiazolidinedione rosiglitazone can effectively improve insulin sensitivity in diabetic patients. GPCRs are one of the target enzymes that are important proteins in cell signaling and have so far been found as therapeutic drugs. The total number of targets is about 500, and GPCR targets account for the vast majority of receptors therein. In recent years, indications for targeting GPCR drugs are expanding from traditional areas such as allergies, hypertension, anesthesia and schizophrenia to new areas such as obesity. An ion channel is a pore-forming protein that traverses the channel by allowing an ion of a particular type to rely on an electrochemical gradient. ICs are small pores in the cell membrane that allow ions to enter and exit the cell. Therefore, most of them have become the targets of some mainstream drugs. Enzymes are macromolecular biocatalysts. Some common drugs use enzymes as targets, and some effects on enzymes such as inhibition, induction, activation or reactivation are exerted. In addition, drugs like this are mostly enzyme inhibitors. According to statistics, half of the top 20 drugs in the world are enzyme inhibitors. It is worth noting that some drugs are enzymes themselves, such as pepsin and trypsin.

Each dataset contains three matrices, **Y**, **S**_**d**_ and **S**_**t**_. Matrix **Y** represents the drug-target interactions. It is worth noting that this matrix is an adjacency matrix. If it is known that the drug *d*_*i*_ is related to the target *t*_*j*_, *Y*_*ij*_ is 1, otherwise *Y*_*ij*_ is 0. The matrix **S**_**d**_ represents the chemical pairing structural similarity [19] and the matrix **S**_**t**_ represents the genome sequence similarity of the target pair [20]. Table 1 lists the specific information for the four datasets. More information about the datasets are published in https://github.com/cuizhensdws/L21-GRMF.

**Table 1:** Drugs, Targets, and Interactions in Each Dataset

Datasets	NR	GPCR	IC	E
Drugs	54	223	210	445
Targets	26	95	204	664
Interactions	90	635	1476	2923

### Cross-validation experiments

We compare the existing matrix decomposition methods CMF (Collaborative matrix factorization), GRMF (Graph regularized matrix factorization), WGRMF (Weighted graph regularized matrix factorization) and our proposed method and compare WKNKN preprocessing on these methods. We use cross-validation experiments on these methods. In this paper, we use a ten-fold cross-validation (CV). The original dataset **Y** is divided into ten subsets, each of which is tested once and the rest as a training set. The cross-validation is repeated five times, one subset is selected each time as a test set, and the average cross-validation recognition accuracy rate of five times is taken as a result.

To verify the effect of the prediction, we use the evaluation index which has been widely used before, the AUPR (Area under the Precision-Recall curve) [21]. There is also an evaluation scale called AUC (Area under the receiver operating characteristic curve). We can use this method when forecasting. In our experiments, ten AUPR values are calculated for each ten-fold cross-validation, an average is obtained and we repeat five times, so we take the average of the five AUPRs as the final result [22]. In general, the AUPR value is less than the AUC value. The AUPR value is above 0.3, so the experimental results are reasonable.

**Fig. 1:**
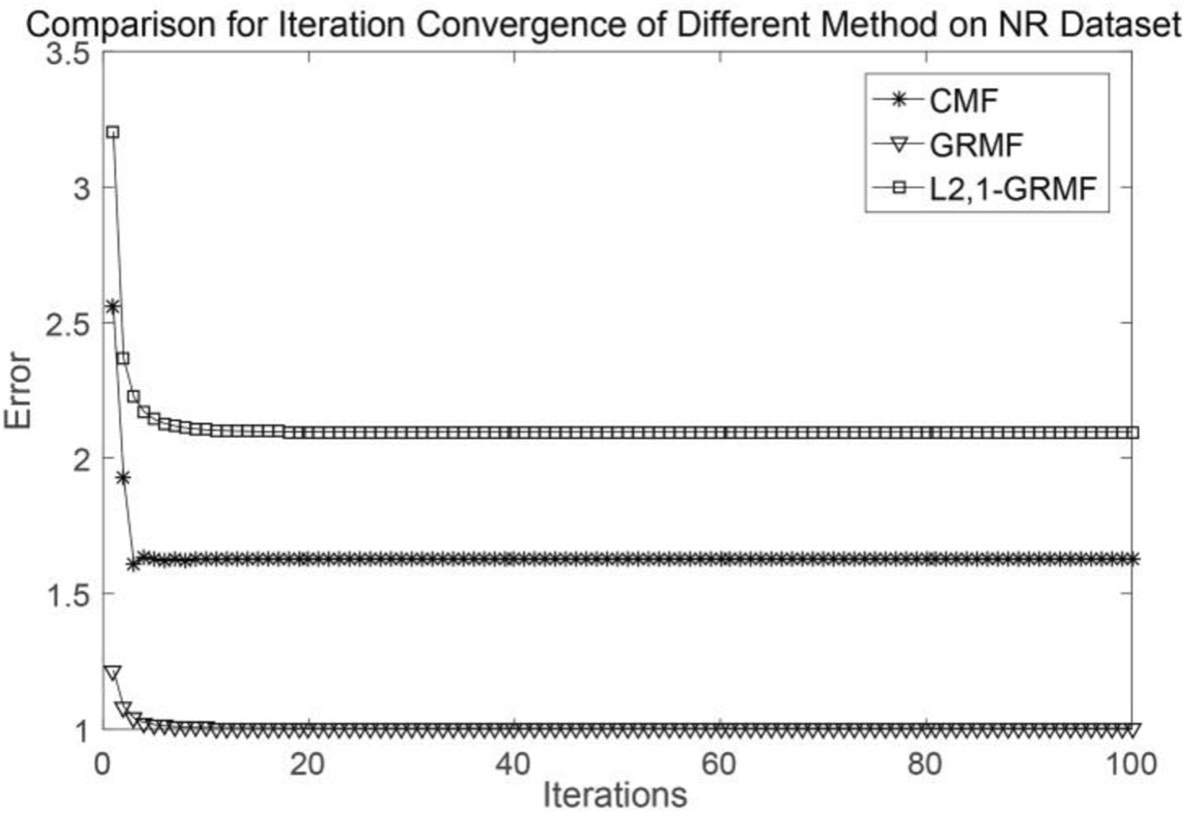
Comparison of convergence about three methods on the NR dataset

**Fig. 2:**
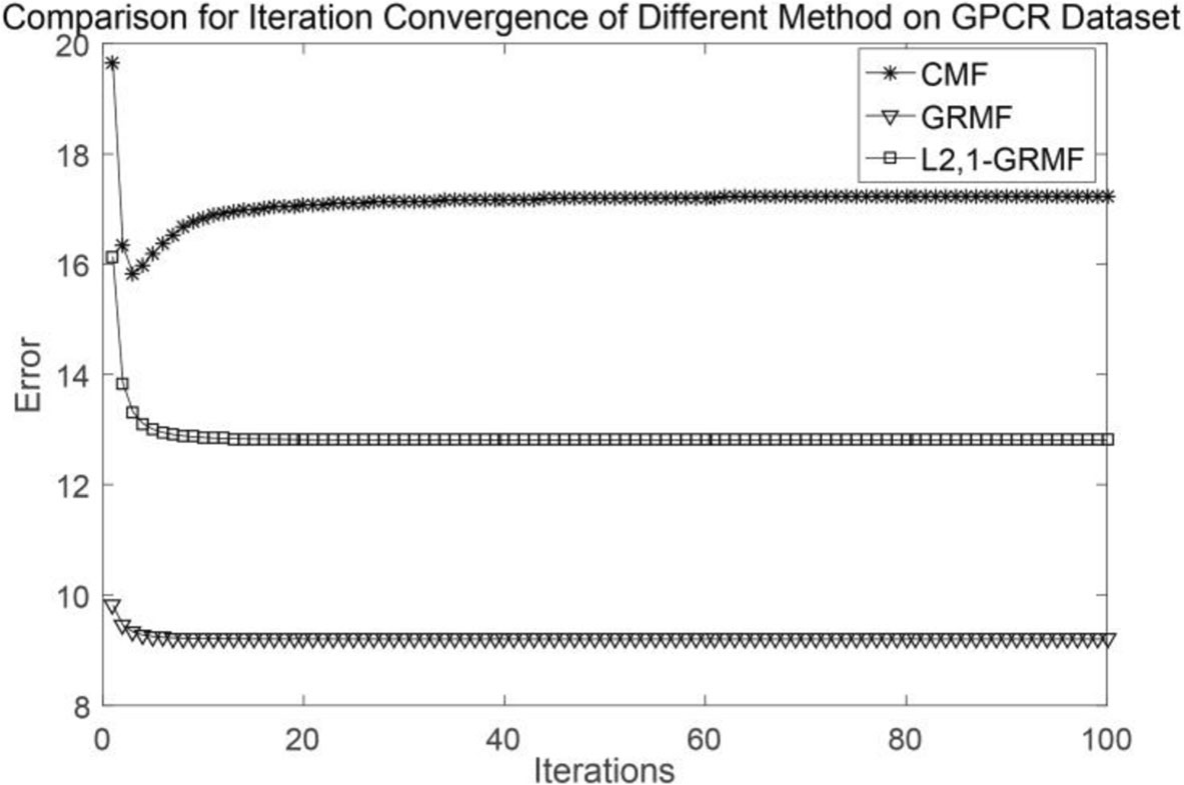
Comparison of convergence about three methods on the GPCR dataset

We test two aspects [23], one is CVd which is based on the drug-interaction profiles and the other is CVt, which is based on the target-interaction profiles. CVd is used to test the ability to predict new drugs, CVt is used to test the ability to predict new targets. In addition, we perform a convergence analysis of each method using the NR and GPCR datasets as examples, and each method is subjected to 100 iterations. When the number of iterations is about 20, our method achieves convergence. It is worth noting that we have different tolerances for errors, considering the size and type of the datasets. Generally speaking, as long as the error is within a reasonable range, this is acceptable. Figures 1 and 2 show the convergence of different methods on the NR and GPCR datasets, respectively.

#### Interaction prediction under CVd

**Fig. 3:**
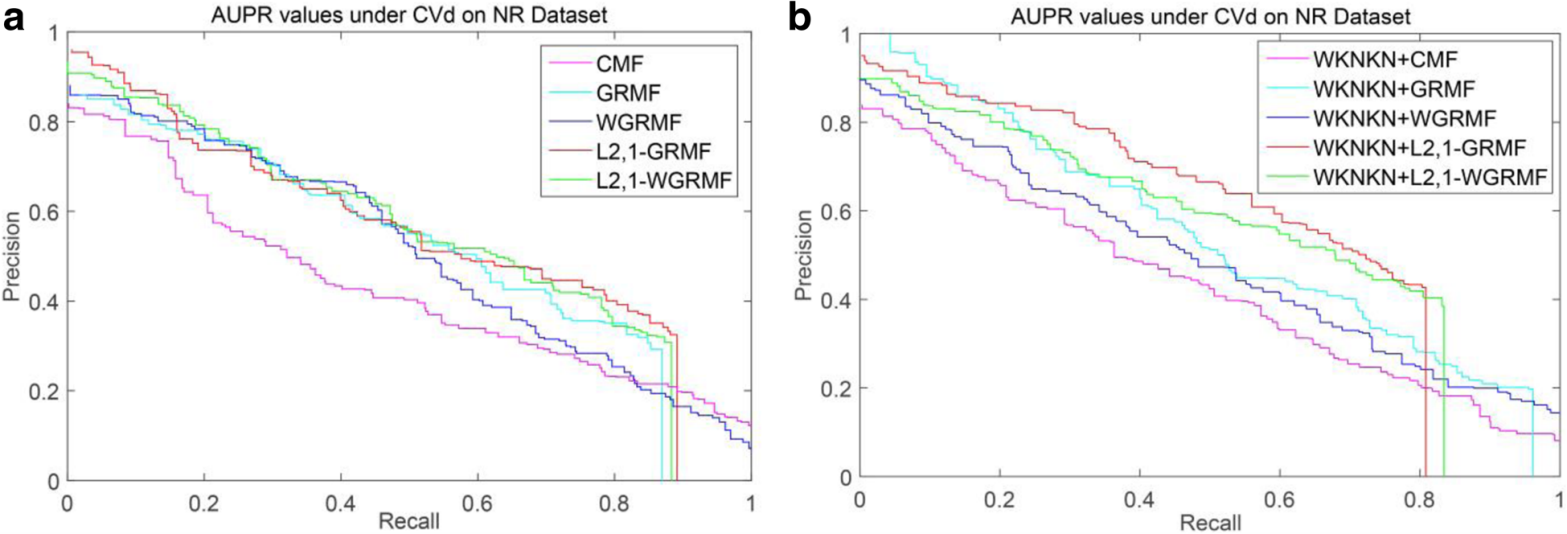
PR curves for different methods are plotted together, providing a visual comparison between their prediction performances. The PR curves on the CVd side of each method on the NR dataset. **a** WKNKN is not used, the PR curves for each method. **b** WKNKN is used, the PR curves for each method

Table 2 lists the experimental results at CVd. And Standard deviations are given in parentheses. Under the NR dataset, the L_2,1_-GRMF (L_2,1_-norm Graph regularized matrix factorization) method is superior to the GRMF method and is almost the same as the GRMF method after adding the WKNKN. Importantly, our improved method L_2,1_-GRMF, with the addition of WKNKN, has seen significant improvements. Moreover, after adding the weight matrix to L_2,1_-GRMF and using WKNKN, the accuracy of prediction is also improved. Figure 3 shows the PR curves on the CVd side of each method on the NR dataset.

**Table 2:** AUPR Results for Interaction Prediction Under CVd

Methods	NR	GPCR	IC	E
CMF	0.482(0.034)	0.406(0.008)	0.350(0.008)	0.375(0.007)
GRMF	0.517(0.025)	0.369(0.011)	0.341(0.016)	0.349(0.012)
WGRMF	0.520(0.025)	0.408(0.010)	0.364(0.018)	0.404(0.014)
L_2,1_-GRMF	0.543(0.034)	0.373(0.011)	0.345(0.012)	0.346(0.013)
L_2,1_-WGRMF	0.542(0.024)	0.400(0.010)	0.370(0.016)	0.408(0.013)
WKNKN+CMF	0.515(0.032)	0.409(0.010)	0.350(0.014)	0.385(0.004)
WKNKN+GRMF	0.542(0.028)	0.404(0.011)	0.356(0.014)	0.390(0.010)
WKNKN+WGRMF	0.528(0.033)	0.410(0.012)	0.369(0.017)	0.401(0.013)
WKNKN+L_2,1_-GRMF	0.573(0.011)	0.394(0.007)	0.356(0.012)	0.386(0.013)
WKNKN+L_2,1_-WGRMF	0.544(0.026)	0.394(0.012)	0.374(0.016)	0.385(0.007)

**Fig. 4:**
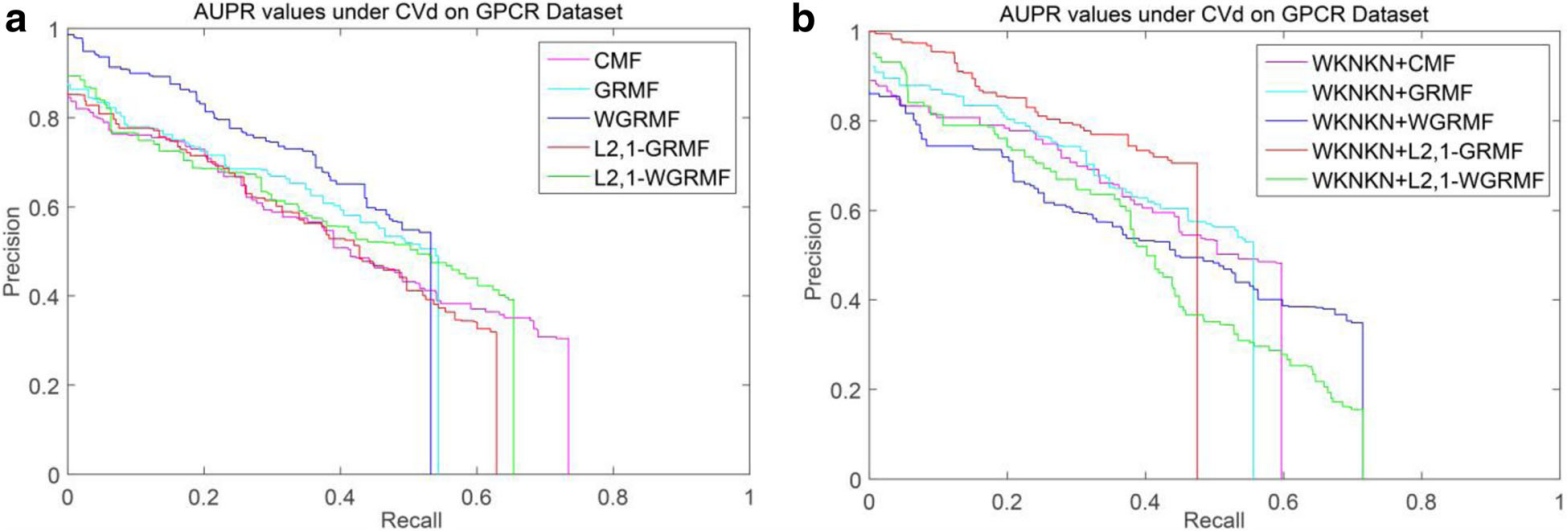
PR curves for different methods are plotted together, providing a visual comparison between their prediction performances. The PR curves on the CVd side of each method on the GPCR dataset. **a** WKNKN is not used, the PR curves for each method. **b** WKNKN is used, the PR curves for each method

**Fig. 5:**
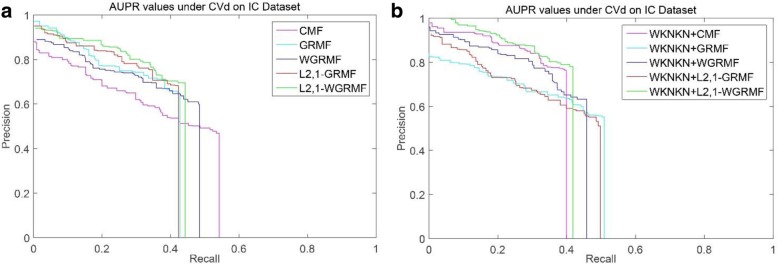
PR curves for different methods are plotted together, providing a visual comparison between their prediction performances. The PR curves on the CVd side of each method on the IC dataset. **a** WKNKN is not used, the PR curves for each method. **b** WKNKN is used, the PR curves for each method

**Fig. 6:**
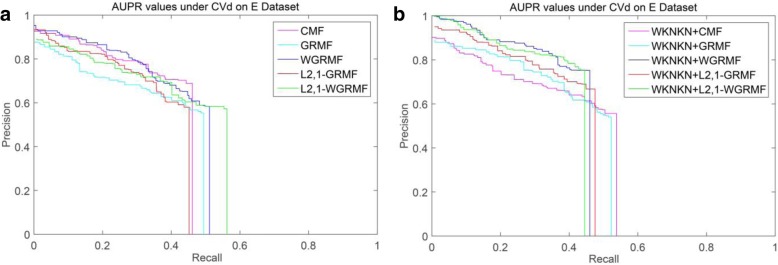
PR curves for different methods are plotted together, providing a visual comparison between their prediction performances. The PR curves on the CVd side of each method on the E dataset. **a** WKNKN is not used, the PR curves for each method. **b** WKNKN is used, the PR curves for each method

However, on the GPCR dataset, we run our method and find that it is not outperform the previous method, and initially estimate that there is a problem with the dataset itself. Figure 4 shows the PR curves on the CVd side of each method on the GPCR dataset. We observe that using the weight matrix when performing CVd experiments is higher than the AUPR value obtained without using the weight matrix. In addition, the L_2,1_-WGRMF (Weighted L_2,1_-norm graph regularized matrix factorization) method using WKNKN is superior to any other method in the IC dataset, slightly better than the WGRMF method using WKNKN. Figure 5 shows the PR curves on the CVd side of each method on the IC dataset. In the E dataset, the best method is L_2,1_-WGRMF but the AUPR score drops instead after applying WKNKN. In other words, in the E dataset, the preprocessing step will actually have a negative effect on the forecast result. Figure 6 shows the PR curves on the CVd side of each method on the E dataset. In general, not all methods use WKNKN to improve AUPR scores, which have a positive effect on most datasets and negative effects on some datasets. In practice, the negative impact of the WKNKN method is unavoidable on some datasets. One important reason is that the WKNKN method assigns an inaccurate value to the 0 element of the matrix **Y** on the E dataset. When we add the L_2,1_-GRMF method to make more accurate predictions, these inaccurate values will reduce the prediction accuracy.

#### Interaction prediction under CVt

We can see in Table 3 that under most datasets, the AUPR value of CVt is generally higher than the AUPR value of CVd. This shows that hiding the interactions of the target can still get a better prediction result. But hiding the drug interactions and the prediction result will be greatly reduced. And standard deviations are given in parentheses. It is worth noting that in most datasets, the CMF method has lower AUPR values than any other method, and its AUPR value is far less than our method, especially in the NR dataset.

**Table 3:** AUPR Results for Interaction Prediction Under CVt

Methods	NR	GPCR	IC	E
CMF	0.379(0.020)	0.540(0.028)	0.751(0.014)	0.740(0.014)
GRMF	0.423(0.032)	0.567(0.027)	0.745(0.008)	0.763(0.020)
WGRMF	0.423(0.017)	0.574(0.027)	0.801(0.008)	0.801(0.018)
L_21_-GRMF	0.465(0.056)	0.607(0.020)	0.823(0.012)	0.804(0.021)
L_2,1_-WGRMF	0.425(0.023)	0.603(0.026)	0.801(0.007)	0.802(0.016)
WKNKN+CMF	0.434(0.029)	0.557(0.021)	0.742(0.015)	0.772(0.014)
WKNKN+GRMF	0.500(0.028)	0.615(0.023)	0.815(0.010)	0.807(0.016)
WKNKN+WGRMF	0.446(0.015)	0.585(0.027)	0.799(0.007)	0.798(0.018)
WKNKN+L_2,1_-GRMF	0.519(0.038)	0.617(0.024)	0.826(0.008)	0.799(0.016)
WKNKN+L_2,1_-WGRMF	0.457(0.032)	0.548(0.021)	0.799(0.012)	0.791(0.014)

## Discussion

**Fig. 7:**
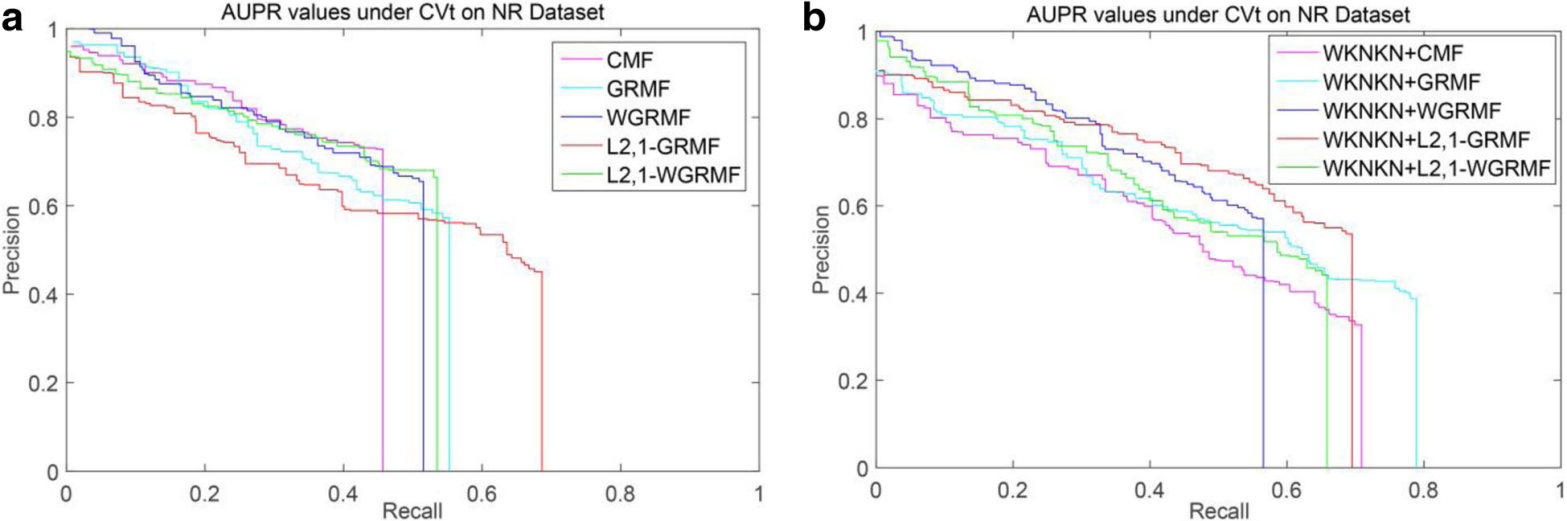
PR curves for different methods are plotted together, providing a visual comparison between their prediction performances. The PR curves on the CVt side of each method on the NR dataset. **a** WKNKN is not used, the PR curves for each method. **b** WKNKN is used, the PR curves for each method

**Fig. 8:**
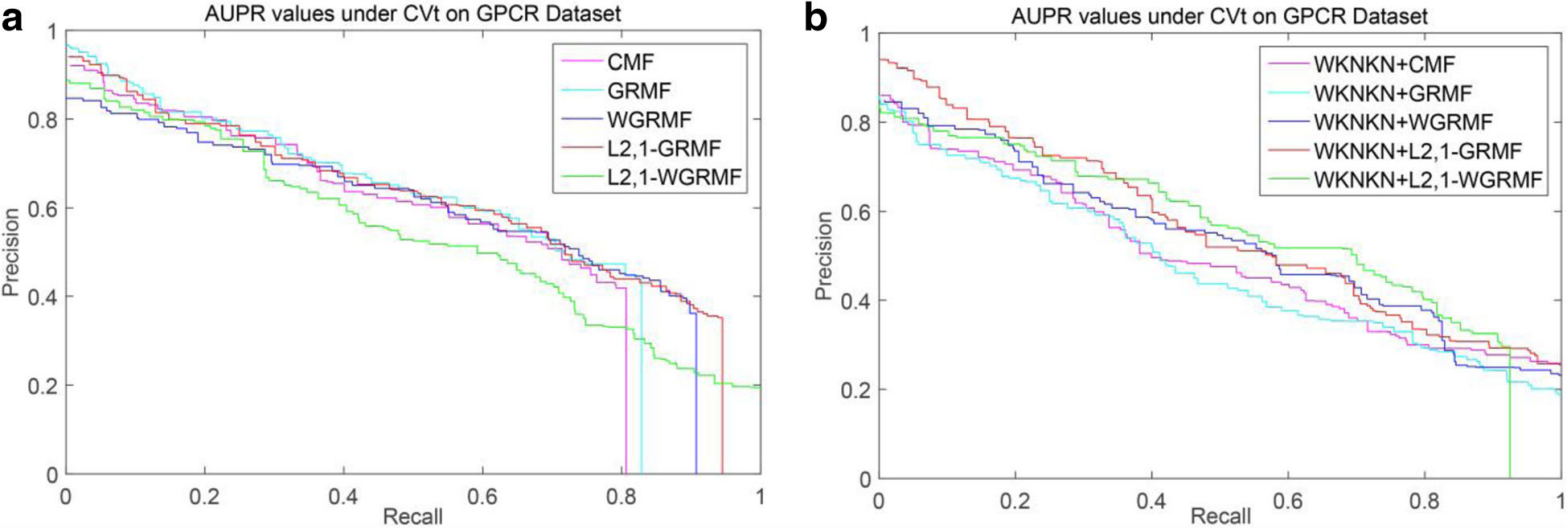
PR curves for different methods are plotted together, providing a visual comparison between their prediction performances. The PR curves on the CVt side of each method on the GPCR dataset. **a** WKNKN is not used, the PR curves for each method. **b** WKNKN is used, the PR curves for each method

**Fig. 9:**
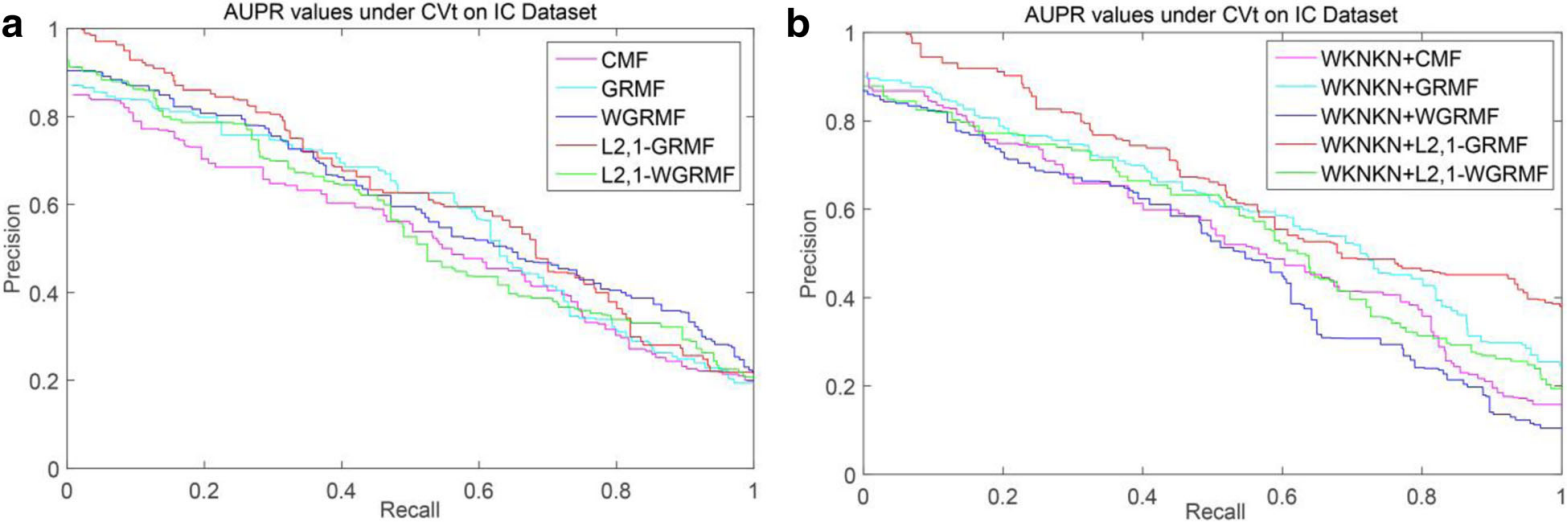
PR curves for different methods are plotted together, providing a visual comparison between their prediction performances. The PR curves on the CVt side of each method on the IC dataset. **a** WKNKN is not used, the PR curves for each method. **b** WKNKN is used, the PR curves for each method

**Fig. 10:**
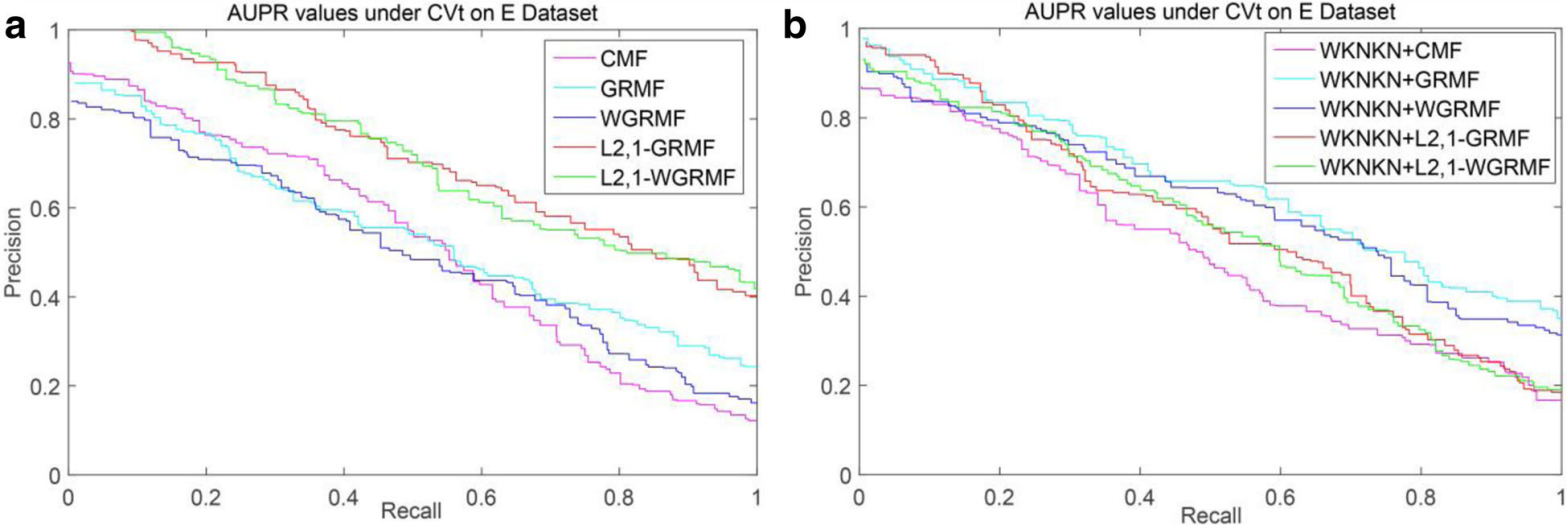
PR curves for different methods are plotted together, providing a visual comparison between their prediction performances. The PR curves on the CVt side of each method on the E dataset. **a** WKNKN is not used, the PR curves for each method. **b** WKNKN is used, the PR curves for each method

Among the NR, GPCR and IC datasets, the superior methods are the L_2,1_-GRMF method using the preprocessing steps, and our improved method has some improvement on all three datasets. Figures 7, 8, 9 and 10 show the PR curves on the CVt side of each method on the NR, GPCR, IC and E datasets, respectively. On the E dataset, it is still the best GRMF method. We can also see that some instances are ignored after using the weight matrix, whereas the GRMF method does not use the weight matrix **W**. Therefore, based on the previous conclusions, the information of the target is more important than the information of the drug. Therefore, using the GRMF method, the AUPR value is higher than the AUPR value using WGRMF.

On most datasets, the L_2,1_-norm does play a key role in predicting the results. The L_2,1_-norm can provide a sparse solution for the final result. Compared with the CMF method, the L_2,1_-norm also promotes the final convergence. Therefore, the overall performance of the L_2,1_-GRMF method and L_2,1_-WGRMF is superior to other methods.

### Case study

In this section, we conduct a simulation experiment. First, we erase some of the known drug targets in the original dataset. That is, those elements that are originally 1 in the original matrix become 0. This process is performed randomly by the computer. In the second step, we perform the experiment. We examine the results of the experiment and see if the erased condition is successfully predicted.

The experimental procedure we implement is that in the NR dataset, ten drugs with the interaction of the target estrogen receptor alpha (KEGG ID: hsa2099) are removed. This target is the main cause of breast cancer. After the experiment is done, we count the experimental results. We predict five of the hidden interactions. At the same time, we also predict a portion of new drugs and take the most reliable top five new drugs stated in Table 4. Among them, the sixth drug Testosterone is the drug with the highest correlation with this target.

**Table 4:** Predicted Drugs for estrogen receptor alpha, NR Dataset

Rank	Drug	Drug ID
1	Progesterone	D00066
2	Estrone	D00067
3	Ethinylestradiol	D00554
4	Etodolac	D00315
5	Ethynodiol diacetate	D01294
6	Testosterone	D00075
7	Budesonide	D00246
8	Isotretinoin	D00348
9	Mometasone furoate	D00690
10	Paricalcitol	D00930

In IC dataset, for the drug Diazoxide (KEGG ID: D00294), a blood pressure lowering drug. We also use a similar approach. Before using the L_2,1_-GRMF method, we eliminate twenty of them in the matrix **Y**. Because the GPCR dataset is larger than the NR dataset and there are many targets associate with this drug, we have removed twenty interactions here. After conducting simulation experiments, we successfully predicted twelve known targets and eight new targets. We then list the top twenty targets in Table 5. The first 12 are known targets and the remaining part is our prediction of a new target.

**Table 5:** Predicted Targets for Diazoxide, IC Dataset

Rank	Target	Target ID
1	potassium voltage-gated channel subfamily J member 16	hsa3773
2	potassium voltage-gated channel subfamily A member regulatory beta subunit 1	hsa7881
3	potassium voltage-gated channel subfamily J member 15	hsa3772
4	potassium voltage-gated channel modifier subfamily S member 2	hsa3788
5	potassium voltage-gated channel subfamily H member 5	hsa27133
6	potassium voltage-gated channel subfamily D member 1	hsa3750
7	glutamate ionotropic receptor AMPA type subunit 1	hsa2890
8	potassium voltage-gated channel subfamily D member 3	hsa3752
9	potassium calcium-activated channel subfamily N member 4	hsa3783
10	potassium voltage-gated channel subfamily H member 1	hsa3756
11	potassium calcium-activated channel subfamily N member 3	hsa3782
12	potassium voltage-gated channel subfamily D member 2	hsa3751
13	chloride voltage-gated channel 2	hsa1181
14	calcium voltage-gated channel auxiliary subunit beta 4	hsa785
15	sodium channel epithelial 1 gamma subunit	hsa6340
16	ryanodine receptor 3	hsa6263
17	cholinergic receptor nicotinic delta subunit	hsa1144
18	solute carrier family 6 member 4	hsa6532
19	sodium voltage-gated channel alpha subunit 3	hsa6328
20	sodium voltage-gated channel alpha subunit 9	hsa6335

For these two cases, the similarity of the estrogen receptor alpha to its nearest neighbor target is less than 0.02 in the matrix **S**^**t**^. In the matrix **S**^**d**^, the similarity of Diazoxide to its nearest neighbor is 0.3, which is also quite low. Therefore, we are more difficult to make predictions. Thus, this shows that our proposed L_2,1_-GRMF method is excellent and reliable results can be obtained when predicting some challenging drugs and targets. Of course, there are still some limitations to the two methods proposed. If we add a weight matrix, the time required for the experiment will multiply. Compared with other methods, our time complexity is relatively high. In addition, the method does not predict new drugs and new targets without any interaction.

## Conclusions

In this paper, we propose two improved matrix decomposition methods, L_2,1_-GRMF and L_2,1_-WGRMF. Both methods are used to predict drug-target interactions. We use cross-validation to calculate AUPR values and predict on the drug side (CVd) and the target side (CVt), respectively. We compare them with the most advanced matrix factorization methods currently available. In most cases, our improved methods can provide the best results, which means that the predictive performance is improved with the use of the L_2,1_-norm.

WKNKN preprocessing steps are used to help the experimental results. In addition, it can also be used as an independent method to predict the interactions of drug-target. Considering that the dimensions of the data are relatively small, so the drug-target interactions contained in each dataset are also limited. And our approach applies to these datasets.

In the future, we expect more and more known interaction of drug targets will be found, providing more valuable datasets for our prediction. We will explore more effective prediction methods to solve drug-target interaction problems. For example, we can use matrix factorization of hyper-graph method to improve the reliability of predictive interactions.

## Methods

### CMF

Co-matrix factorization is an effective method to predict the interactions of drug-target [15]. The objective function of CMF method is

1 $$ {\min}_{\mathbf{A},\mathbf{B}}={\left\Vert \mathbf{Y}-{\mathbf{A}\mathbf{B}}^{\mathrm{T}}\right\Vert}_F^2+{\lambda}_l\left({\left\Vert \mathbf{A}\right\Vert}_F^2+{\left\Vert \mathbf{B}\right\Vert}_F^2\right)+{\lambda}_d{\left\Vert {\mathbf{S}}^{\mathbf{d}}-{\mathbf{A}\mathbf{A}}^{\mathrm{T}}\right\Vert}_F^2+{\lambda}_t{\left\Vert {\mathbf{S}}^{\mathbf{t}}-{\mathbf{BB}}^{\mathrm{T}}\right\Vert}_F^2, $$

where **W** represents a weight matrix, *W*_*ij*_ = 1 when *Y*_*ij*_ is known, *W*_*ij*_ = 0 otherwise. Obviously, the last two items of the objective function are regularization terms. We use *L* to represent the objection function in Eq. (1), *a*_*i*_ represents the *i*-th vector of **A**, and *b*_*j*_ represents the *j*-th vector of **B**. Two update rules are used to solve *∂L*/*∂a* = 0 and *∂L*/*∂b* = 0. Finally, the two update rules are executed using least square until convergence:

2 $$ \mathbf{A}=\left(\mathbf{YB}+{\lambda}_d{\mathbf{S}}^{\mathbf{d}}\mathbf{A}\right){\left({\mathbf{B}}^{\mathrm{T}}\mathbf{B}+{\lambda}_l{\mathbf{I}}_{\mathrm{k}}+{\lambda}_d{\mathbf{AA}}^{\mathrm{T}}\right)}^{-1}, $$

3 $$ \mathbf{B}=\left({\mathbf{Y}}^{\mathrm{T}}\mathbf{A}+{\lambda}_t{\mathbf{S}}^{\mathbf{t}}\mathbf{B}\right){\left({\mathbf{A}}^{\mathrm{T}}\mathbf{A}+{\lambda}_l{\mathbf{I}}_{\mathrm{k}}+{\lambda}_t{\mathbf{B}}^{\mathrm{T}}\mathbf{B}\right)}^{-1}. $$

In summary, after the potential feature matrices **A** and **B** are updated, the predicted score matrix can be obtained by multiplying **A** and **B**. This predicted score matrix can be used to predict new drug-target interactions by comparing with the original drug-target interactions matrix **Y**.

### GRMF

In the GRMF method, the benefits of regularization items is that it can avoid over-fitting [20]. The objective function of GRMF is as follows:

4 $$ {\min}_{\mathbf{A},\mathbf{B}}={\left\Vert \mathbf{Y}-{\mathbf{A}\mathbf{B}}^{\mathrm{T}}\right\Vert}_F^2+{\lambda}_l\left({\left\Vert \mathbf{A}\right\Vert}_F^2+{\left\Vert \mathbf{B}\right\Vert}_F^2\right)+{\lambda}_d\mathrm{Tr}\left({\mathbf{A}}^{\mathrm{T}}\overset{\sim }{{\mathbf{L}}_{\mathbf{d}}}\mathbf{A}\right)+{\lambda}_t\mathrm{Tr}\left({\mathbf{B}}^{\mathrm{T}}\overset{\sim }{{\mathbf{L}}_{\mathbf{t}}}\mathbf{B}\right), $$

Then, matrix **A** and **B** are initialized. The SVD (singular value decomposition) method is used to decompose matrix **Y** ∈ *R*^*n* × *m*^ into **U** ∈ *R*^*n* × *k*^, **S**_**k**_ ∈ *R*^*k* × *k*^, and **V** ∈ *R*^*n* × *k*^. In matrix **Y**, the largest possible number of singular values is min(*n*, *m*), so *k* max  = min(*n*, *m*). Finally, the square root of **S**_**k**_ can be obtained, where $\mathbf{A}={\mathbf{US}}_{\mathbf{k}}^{1/2}$, $\mathbf{B}={\mathbf{VS}}_{\mathbf{k}}^{1/2}$.

Next, the least square method is used to update **A** and **B**. This objective function in Eq. (4) can be replaced by *L*. These two update rules are used to solve *∂L*/*∂a* = 0 and *∂L*/*∂b* = 0. Finally, the two update rules are executed by using least square until convergence.

### WGRMF

Like CMF, the weight matrix **W** in WGRMF is the same as **W** in CMF. Behind the weight matrix, either to prevent unknown interactions, the purpose is to help find the latent feature matrix **A** and **B**. The objective function of WGRMF method is as follows

5 $$ {\min}_{\mathbf{A},\mathbf{B}}={\left\Vert \mathbf{W}\odot \left(\mathbf{Y}-{\mathbf{A}\mathbf{B}}^{\mathrm{T}}\right)\right\Vert}_F^2+{\lambda}_l\left({\left\Vert \mathbf{A}\right\Vert}_F^2+{\left\Vert \mathbf{B}\right\Vert}_F^2\right)+{\lambda}_d\mathrm{Tr}\left({\mathbf{A}}^{\mathrm{T}}\overset{\sim }{{\mathbf{L}}_{\mathbf{d}}}\mathbf{A}\right)+{\lambda}_t\mathrm{Tr}\left({\mathbf{B}}^{\mathrm{T}}\overset{\sim }{{\mathbf{L}}_{\mathbf{t}}}\mathbf{B}\right). $$

This objective function in Eq. (5) can be replaced by *L*, where *a*_*i*_ represents the *i*-th vector of **A**, and *b*_*j*_ represents the *j*-th vector of **B**. These two update rules are used to solve *∂L*/*∂a* = 0 and *∂L*/*∂b* = 0. Finally, the two update rules are executed by using least square until convergence. However, it is worth noting that the update rules here are not the same as the update rules in GRMF. In GRMF, the rules are matrix updates, but in WGRMF the rules are row updates.

### Our proposed methods

**Fig. 11:**
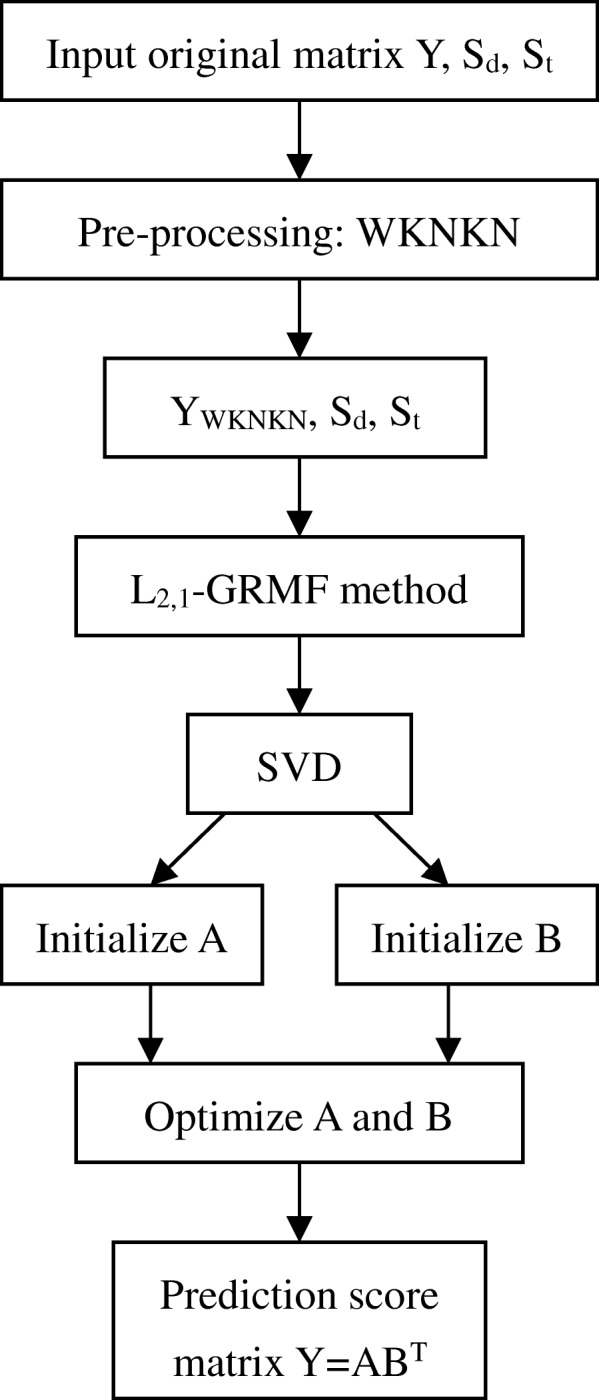
A brief flow chart of the L_2,1_-GRMF method. It includes the process of inputting the original datasets to the final generation of the predicted score matrix

Here, our improved approach is used to solve the prediction of drug-target interactions problem. WKNKN (weighted K nearest known neighbors) [20] as a preprocessing step is used to solve unknown missing value problems. Two methods are proposed, Graph Regularization Matrix factorization based on L_2,1_-norm, and a variant called L_2,1_-WGRMF, both of which are used to predict drug-target interactions. Figure 11 shows a flow chart of the proposed method.

### L_2,1_-GRMF

#### Sparsification of the drug similarity matrix and target similarity matrix

Graph regularization terms are used to fully consider the internal structure of the similarity matrix **S**^**d**^ and **S**^**t**^. In addition, the graph regularization terms can keep the internal structure of the matrices unchanged. We derive a *p*-nearest neighbor graph from each drug and target similarity matrix [24] **S**^**d**^ and **S**^**t**^ in this work. Therefore, given a drug similarity matrix **S**^**d**^, a *p*-nearest neighbor graph [25] *N* can be generated as

6 $$ {\forall}_{i,j},\kern0.5em {N}_{ij}=\left\{\begin{array}{l}1,\kern1.4em j\in {N}_p(i)\kern0.3em \&\kern0.3em i\in {N}_p(j)\kern0.1em \\ {}0,\kern1.3em j\notin {N}_p(i)\kern0.3em \&\kern0.3em i\notin {N}_p(j)\\ {}0.5,\kern0.7em otherwise,\end{array}\right. $$

where *N* is used to sparsify the matrix **S**^**d**^, which can be written as

7 $$ {\forall}_{i,j},\kern0.3em \hat{S}={N}_{ij}{S}_{ij}^d. $$

This result is for a sparse drug similarity matrix. Similarly, the target similarity matrix **S**^**t**^ can be obtained in the same way. We use the Euclidean distance to calculate the nearest neighbor. In general, Euclidean distance will give better results because it represents the true distance.

Graph regularization helps to facilitate the study the manifold from learning drugs and target spaces. In the original space, there are points that are close to each other, and when the manifold learning is performed, the points are also close to each other in learning.

#### Low-rank approximation

The idea of low rank approximation (LRA) is applied to GRMF [26]. It decomposes the target matrix **Y** into two low-rank latent feature matrices **A** and **B**, i.e., **Y** ≈ **AB**^T^ [27]. And the objective function of GRMF can be written as the following optimization problem:

8 $$ {\min}_{\mathbf{A},\mathbf{B}}={\left\Vert \mathbf{Y}-{\mathbf{A}\mathbf{B}}^{\mathrm{T}}\right\Vert}_F^2, $$

where ‖⋅‖_*F*_ is Frobenius norm. In addition, the number of potential features of **A** and **B** is represented by *k*.

#### Regularization

In general, the Tikhonov and graph regularization terms can be used to avoid over-fitting and enhance generalization capability. Here is the objective function of L_2,1_-GRMF:

9 $$ {\min}_{\mathbf{A},\mathbf{B}}={\left\Vert \mathbf{Y}-{\mathbf{A}\mathbf{B}}^{\mathrm{T}}\right\Vert}_F^2+{\lambda}_l\left({\left\Vert \mathbf{A}\right\Vert}_F^2+{\left\Vert \mathbf{B}\right\Vert}_F^2\right)+{\lambda}_l{\left\Vert \mathbf{B}\right\Vert}_{2,1}+{\lambda}_d\sum \limits_{i,r=1}^n\hat{S_{ir}^d}{\left\Vert {a}_i-{a}_r\right\Vert}^2+{\lambda}_t\sum \limits_{j,q=1}^m\hat{S_{jq}^t}{\left\Vert {b}_j-{b}_q\right\Vert}^2, $$

where *λ*_*l*_, *λ*_*d*_ and *λ*_*t*_ are positive parameters, *a*_*i*_ is the *i*-th rows of **A**, and *b*_*j*_ is the *j*-th rows of **B**, *n* is the number of drugs, and *m* is the number of targets. The first term is an approximate model of the matrix **Y**. The second term is the Tikhonov regularization. Its main purpose is to minimize the norms of **A**, **B**. The third term is the L_2,1_-norm applied on **B** to increase the target matrix sparsity and discard unwanted target pairs. Considering that we are more concerned with certain drugs, we use the L_2,1_-norm to sparse the potential feature matrix of the target, so that we can better predict new drugs. However, while the L_2,1_-norm is added to **A**, some of the more important drugs may be lost. The last two terms are graph regularization of drugs and targets, respectively. Moreover, the drug-target model can be rewritten as:

10 $$ {\min}_{\mathbf{A},\mathbf{B}}={\left\Vert \mathbf{Y}-{\mathbf{A}\mathbf{B}}^{\mathrm{T}}\right\Vert}_F^2+{\lambda}_l\left({\left\Vert \mathbf{A}\right\Vert}_F^2+{\left\Vert \mathbf{B}\right\Vert}_F^2\right)+{\lambda}_l{\left\Vert \mathbf{B}\right\Vert}_{2,1}+{\lambda}_d\mathrm{Tr}\left({\mathbf{A}}^{\mathrm{T}}{\mathbf{L}}_{\mathbf{d}}\mathbf{A}\right)+{\lambda}_t\mathrm{Tr}\left({\mathbf{B}}^{\mathrm{T}}{\mathbf{L}}_{\mathbf{t}}\mathbf{B}\right), $$

where Tr(⋅) is the trace of the matrix, ${\mathbf{L}}_{\mathbf{d}}={\mathbf{D}}^{\mathbf{d}}-\hat{{\mathbf{S}}^{\mathbf{d}}}$ is the graph Laplacian for $\hat{{\mathbf{S}}^{\mathbf{d}}}$, ${\mathbf{L}}_{\mathbf{t}}={\mathbf{D}}^{\mathbf{t}}-\hat{{\mathbf{S}}^{\mathbf{t}}}$ is the graph Laplacian for $\hat{{\mathbf{S}}^{\mathbf{t}}}$. Please refer to [28] for more details on rewriting graph regularization. We know that the known normalized Laplacian is better than unknown, so we replace **L**_**d**_ and **L**_**t**_ with $\overset{\sim}{{\mathbf{L}}_{\mathbf{d}}}={\left({\mathbf{D}}^{\mathbf{d}}\right)}^{-1/2}{\mathbf{L}}_{\mathbf{d}}{\left({\mathbf{D}}^{\mathbf{d}}\right)}^{-1/2}$ and $\overset{\sim}{{\mathbf{L}}_{\mathbf{t}}}={\left({\mathbf{D}}^{\mathbf{t}}\right)}^{-1/2}{\mathbf{L}}_{\mathbf{t}}{\left({\mathbf{D}}^{\mathbf{t}}\right)}^{-1/2}$. The function can be written as:

11 $$ {\min}_{\mathbf{A},\mathbf{B}}={\left\Vert \mathbf{Y}-{\mathbf{A}\mathbf{B}}^{\mathrm{T}}\right\Vert}_F^2+{\lambda}_l\left({\left\Vert \mathbf{A}\right\Vert}_F^2+{\left\Vert \mathbf{B}\right\Vert}_F^2\right)+{\lambda}_l{\left\Vert \mathbf{B}\right\Vert}_{2,1}+{\lambda}_d\mathrm{Tr}\left({\mathbf{A}}^{\mathrm{T}}\overset{\sim }{{\mathbf{L}}_{\mathbf{d}}}\mathbf{A}\right)+{\lambda}_t\mathrm{Tr}\left({\mathbf{B}}^{\mathrm{T}}\overset{\sim }{{\mathbf{L}}_{\mathbf{t}}}\mathbf{B}\right). $$

We use the minimization of the objective function to predict the outcome of the interactions, but this could lead to unsatisfactory results. Because there are many zeros that have not been found. Therefore, we use WKNKN pre-processing method to solve this problem.

#### Initialization of **A** and **B**

For the input matrix **Y**, SVD (Singular Value Decomposition) method is used to obtain the initial value of matrix **A** and matrix **B**:

12 $$ \left[\mathbf{U},\mathbf{S},\mathbf{V}\right]= SVD\left(\mathbf{Y},k\right),\mathbf{A}={\mathbf{US}}_{\mathbf{k}}^{\mathbf{1}/\mathbf{2}},\mathbf{B}={\mathbf{VS}}_{\mathbf{k}}^{\mathbf{1}/\mathbf{2}}. $$

Among them, **S**_**k**_ is a diagonal matrix and contains the *k* largest singular values. In matrix **Y**, the number of singular values is *k*_max_ = min(*n*, *m*). According to the SVD method, *k*_max_ is the maximum possible number.

#### Optimization algorithm

In this paper, we can update **A** and **B** by using the least square method. Let the partial derivative of **A** be equal to 0, the partial derivative of **B** be equal to 0, the objective function in Eq. (11) can be replaced by *L*, that is, *∂L*/*∂***A** = 0 and *∂L*/*∂***B** = 0. The two update rules are executed by using least square until convergence. When we perform the L_2,1_-GRMF method, *λ*_*l*_, *λ*_*d*_ and *λ*_*t*_ are determined by the cross-validation on the training set to the optimal parameter values. We use grid search, *λ*_*l*_ ∈ {2^−2^, 2^−1^, 2^0^, 2^1^}. Then we choose the optimal parameters from this set. Derivation process is as follows:

13 $$ \mathbf{A}=\left(\mathbf{YB}-{\lambda}_d\overset{\sim }{{\mathbf{L}}_{\mathbf{d}}}\mathbf{A}\right){\left({\mathbf{B}}^{\mathrm{T}}\mathbf{B}+{\lambda}_l{\mathbf{I}}_{\mathbf{k}}\right)}^{-1}, $$

14 $$ \mathbf{B}=\left({\mathbf{Y}}^{\mathrm{T}}\mathbf{A}-{\lambda}_t\overset{\sim }{{\mathbf{L}}_{\mathbf{t}}}\mathbf{B}\right){\left({\mathbf{A}}^{\mathrm{T}}\mathbf{A}+{\lambda}_l{\mathbf{I}}_{\mathbf{k}}+{\lambda}_l{\mathbf{DI}}_{\mathbf{k}}\right)}^{-1}, $$

where **D** is a diagonal matrix with the *i*-th diagonal element as *d*_*ii*_ = 1/2‖(**B**)^*i*^‖_2_. The specific algorithm of L_2,1_-GRMF is as follows:

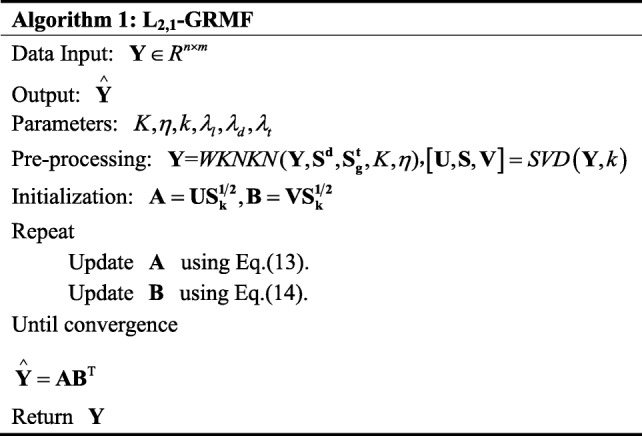



### L_2,1_-WGRMF

A variant of L_2,1_-GRMF, called L_2,1_-WGRMF, is obtained here by adding a weight matrix **W** to the L_2,1_-GRMF. The advantage is that it helps to determine the latent feature matrices **A** and **B** of the drug-target matrix **Y**. So, we write the objective function that contains **W** as follows:

15 $$ {\min}_{\mathbf{A},\mathbf{B}}={\left\Vert \mathbf{W}\odot \left(\mathbf{Y}-{\mathbf{A}\mathbf{B}}^{\mathrm{T}}\right)\right\Vert}_F^2+{\lambda}_l\left({\left\Vert \mathbf{A}\right\Vert}_F^2+{\left\Vert \mathbf{B}\right\Vert}_F^2\right)+{\lambda}_l{\left\Vert \mathbf{B}\right\Vert}_{2,1}+{\lambda}_d\mathrm{Tr}\left({\mathbf{A}}^{\mathrm{T}}{\mathbf{L}}_{\mathbf{d}}\mathbf{A}\right)+{\lambda}_t\mathrm{Tr}\left({\mathbf{B}}^{\mathrm{T}}{\mathbf{L}}_{\mathbf{t}}\mathbf{B}\right). $$

Let objective function be set to *F* such that *∂F*/*∂a*_*i*_ = 0 and *∂F*/*∂b*_*j*_ = 0. The update rules are used to obtain **A** and **B** until convergence

16 $$ {\forall}_i=1\dots n,\kern0.9000001em {a}_i=\left(\sum \limits_{j=1}^m{W}_{ij}{Y}_{ij}{b}_j-{\lambda}_d{\left(\overset{\sim }{{\mathbf{L}}_{\mathbf{d}}}\right)}_{i\ast}\mathbf{A}\right){\left(\sum \limits_{j=1}^m{W}_{ij}{b}_j^{\mathrm{T}}{b}_j+{\lambda}_l{\mathbf{I}}_{\mathbf{k}}\right)}^{-1}, $$

17 $$ {\forall}_j=1\dots m,\kern0.8000001em {b}_j=\left(\sum \limits_{i=1}^n{W}_{ij}{Y}_{ij}{a}_i-{\lambda}_t{\left(\overset{\sim }{{\mathbf{L}}_t}\right)}_{j\ast}\mathbf{B}\right){\left(\sum \limits_{i=1}^n{W}_{ij}{a}_i^{\mathrm{T}}{a}_i+{\lambda}_l{\mathbf{I}}_{\mathbf{k}}+{\lambda}_l{\mathbf{DI}}_{\mathbf{k}}\right)}^{-1}. $$

## Abbreviations

AUPR: Area under the precision-recall curve
CMF: Collaborative matrix factorization method
CV: Cross-validation
GRMF: Graph regularized matrix factorization
L_2,1_-GRMF: L_2,1_-norm Graph regularized matrix factorization
L_2,1_-WGRMF: Weighted L_2,1_-norm graph regularized matrix factorization
LRA: Low rank approximation
SVD: Singular value decomposition
WGRMF: Weighted graph regularized matrix factorization
WKNKN: Weighted K nearest known neighbors
